# Two Strokes in One Patient: An Interesting Case Report

**DOI:** 10.7759/cureus.69089

**Published:** 2024-09-10

**Authors:** Abhay Lune, Apurva Prabhudesai

**Affiliations:** 1 Ophthalmology, Dr. D. Y. Patil Medical College, Hospital and Research Centre, Dr. D. Y. Patil Vidyapeeth (Deemed to be University), Pune, IND

**Keywords:** central retinal artery occlusion (crao), cherry red spot, ocular emergency, ocular stroke, vision

## Abstract

Central retinal arterial occlusion (CRAO) causes a sudden and devastating visual loss. Transient blurring of vision may precede CRAO and is often ignored by the patient, as it may resolve spontaneously without permanent visual loss. However, this can be a warning sign of ischaemia, particularly in individuals with multiple risk factors.

We report a case of a 50-year-old man with CRAO in his left eye. The patient had a history of transient blurring of vision in the same eye 15 days prior, which resolved without intervention. He also had a history of stroke 12 years ago and had been on anticoagulants but discontinued them three years before this episode. This, combined with chronic smoking and poorly controlled diabetes mellitus, likely contributed to a hypercoagulable state, leading to thrombus formation responsible for CRAO. Fundus examination revealed an opaque and oedematous macula with a cherry red spot and blurred optic disc margins. Treatment with ocular massage and paracentesis improved the patient's vision to finger counting at 2 m. The patient was started on aspirin and advised of strict blood sugar control. This case highlights the importance of recognising transient ischaemic symptoms and maintaining long-term anticoagulant therapy to prevent severe complications like CRAO.

## Introduction

Central retinal arterial occlusion (CRAO) is an ophthalmic emergency that causes sudden, severe, and painless loss of vision in the involved eye [1,2]. von Graefe first described CRAO in 1859, and it has been a well-known ophthalmic entity since then [2].

The commonest cause of CRAO is blockage of the central retinal artery due to an embolus or thrombus. An embolus obstructs the central retinal artery at its narrowest point, where it enters the optic nerve sheath. The thrombus usually blocks the central retinal artery at the level of the lamina cribrosa. Other causes include vasculitis, chronic systemic autoimmune diseases, or thrombophilia [3].

Twelve per cent of non-arteritic CRAO (NA‑CRAO) cases have a history of transient ischaemic attack (TIA) before the development of CRAO [3]. Hypertension, diabetes mellitus, age, hyperlipidaemia, obesity, smoking, and alcohol consumption are the main risk factors for CRAO. These are also the same risk factors for stroke and heart disease. Moreover, CRAO and cerebral ischaemic stroke share the same underlying mechanisms [4]. In a study by Hayreh et al., 20% of CRAO patients had diabetes mellitus, and 38% of CRAO patients were chronic smokers [5].

Our patient had a cerebral stroke 12 years ago at a young age, for which he underwent blood investigations, including a lipid profile, showing elevated triglycerides and deranged blood sugar levels. He also reported smoking around 20 cigarettes per day. All these factors may have contributed to the stroke. The patient was started on anticoagulants, anti-diabetic medications, and lipid-lowering drugs. He was also advised to quit smoking. However, he stopped the anticoagulants on his own three years ago and resumed smoking. His diabetes was also not well controlled. All these factors led to a hypercoagulable state, contributing to the current attack of CRAO.

Our case report highlights the fact that multiple risk factors can coexist and contribute to CRAO. The patient was a known diabetic and a chronic smoker. He had a previous history of cerebral stroke and a recent history of TIA in the left eye. All of these factors, whether individually or collectively, are risk factors for the development of CRAO.

## Case presentation

A 50-year-old man reported to the emergency room with complaints of sudden, painless loss of vision in the left eye for a duration of six hours. He provided a history of a similar episode 15 days before, which lasted for approximately a few minutes and resolved spontaneously without any intervention.

The patient had no history of headaches, giddiness, or nausea. He had been a known case of type 2 diabetes mellitus for 12 years and was under treatment (tablet metformin 500 mg and glimepiride 2 mg twice per day). 

The patient had a past history of cerebral stroke 12 years ago, with right-sided paresis of the upper limb. He had been admitted to a hospital in his hometown and undergone blood investigations, including a lipid profile, which revealed that triglycerides were elevated and blood sugar levels were raised. He was a heavy smoker with a history of smoking approximately 20 cigarettes per day. After undergoing routine blood investigations and diagnostic procedures in his hometown, the patient was started on anticoagulants, anti-diabetic medications, and lipid-lowering drugs. He was also advised to cease smoking. The stroke resolved two weeks after starting treatment.

Three years ago, the patient had stopped anticoagulants on his own, without any medical consultation. He had resumed smoking (five to seven cigarettes per day) and did not monitor his blood sugar levels regularly. He also revealed occasional alcohol intake.

Upon examination, the right eye's best corrected visual acuity for distant vision was 6/6, and for near vision, it was N6, with intact colour vision. The left eye's vision was finger counting close to the face and did not improve with the use of a pinhole. Left eye near vision was poor (less than N36), and colour vision was impaired.

Anterior segment examination of the right eye did not reveal any abnormalities. We classified the left eye's pupillary reaction as Grade 1 relative afferent pupillary defect (RAPD), due to weak initial pupillary constriction followed by greater dilation. The rest of the anterior segment did not show any abnormalities. Fundus examination of the right eye showed clear media; the optic disc was within normal limits, with a cup-to-disc ratio of 0.3 and a healthy neuro-retinal rim (NRR). The macula and general fundus were within normal limits. The left eye's macula was opaque and oedematous, and the oedema extended up to the arcade, with a cherry-red spot at the foveal centre that was suggestive of CRAO.

The patient received a digital ocular massage in the emergency room, every 20 seconds for three to four minutes, followed by a massage with a four-mirror lens for four to five minutes. This was followed by anterior chamber paracentesis, which was performed under topical anaesthesia. We started the patient on topical antibiotic eye drops four times a day, non-steroidal anti-inflammatory eye drops twice a day in the left eye, and a tablet of 250 mg of acetazolamide three times a day. After 30 minutes post-paracentesis, the patient's vision improved to finger counting at 2 m in the left eye. The next day, his best corrected visual acuity in the right eye was 6/6 for distant vision and N6 for near vision, while the left eye's visual acuity was finger counting at 2 m for distance and less than N36 for near vision.

Laboratory investigations were performed, which were normal except for raised blood sugar levels (187 mg/dL fasting and 224 mg/dL post-prandial), raised triglycerides, and negative results for autoimmune conditions.

**Figure 1:**
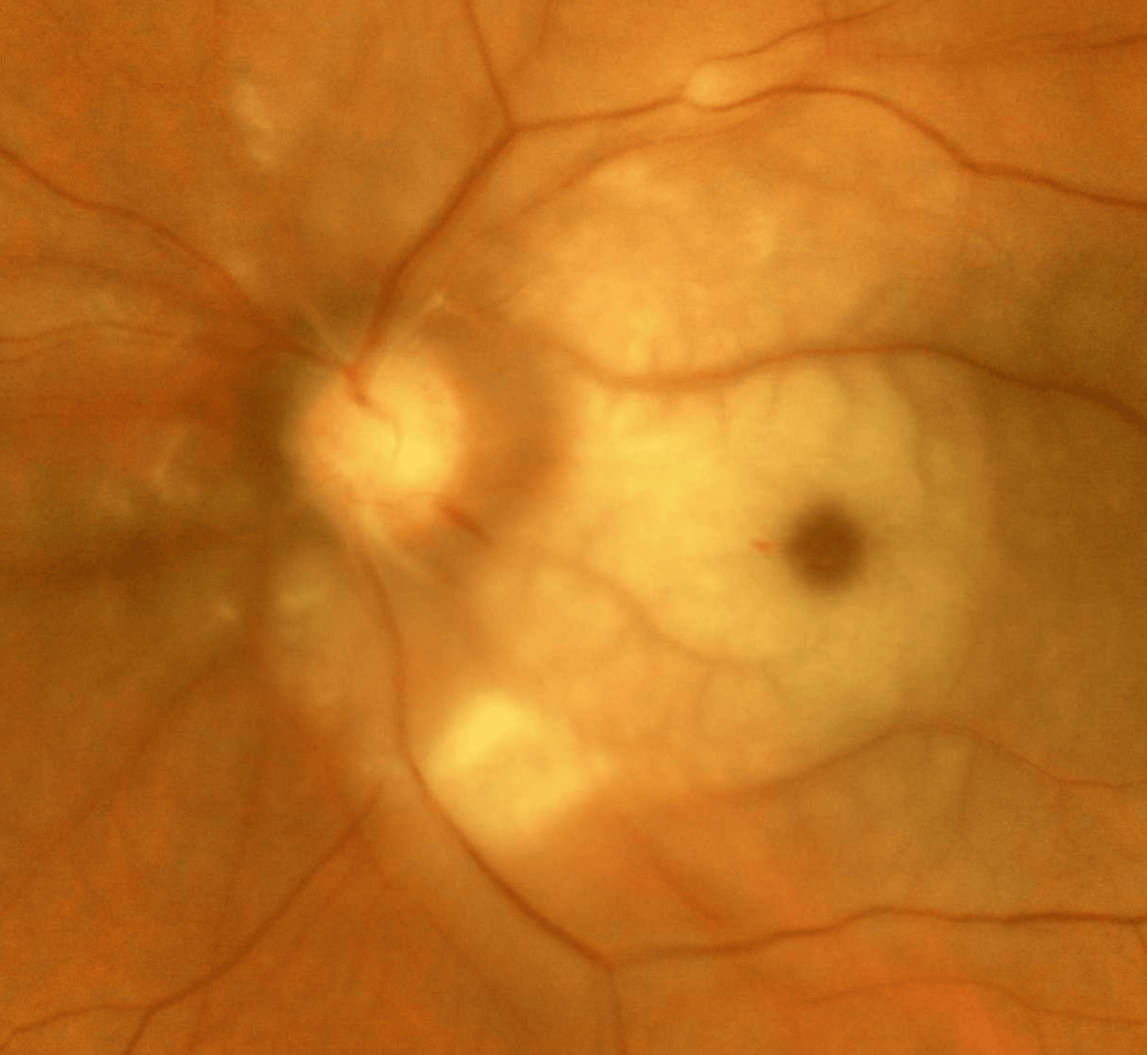
Left eye fundus photography shows blurred disc margin, retinal oedema with cherry-red spot, and splinter haemorrhage at the 5 o'clock disc margin.

Fundus photography was done for documentation of left eye CRAO (Figure 1). The left eye fundus photograph shows blurred disc margins, dense macular oedema, and a cherry-red spot. There is an absence of multiple plaques in the retinal branches. The cilioretinal artery is not seen. A splinter haemorrhage is present at the optic disc margin at the 5 o'clock location. The rest of the retinal vasculature is normal.

Acute ischaemia leads to the opacification of ganglion cells. The density of ganglion cells is maximum at the macula, and hence it appears more opaque compared to the rest of the retina. At the fovea, there is an absence of ganglion cells, and the choroidal vasculature is seen, leading to the appearance of a cherry-red spot. This indicates that the primary cause of occlusion is likely due to a thrombus or emboli, and is localised to the central retinal artery.

**Figure 2:**
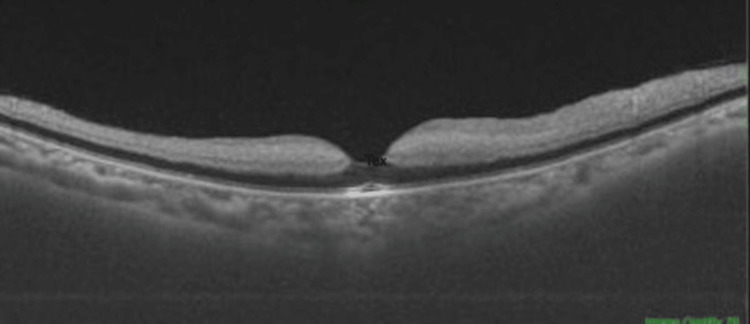
Left eye OCT analysis showed thickening of the inner retinal layers, loss of stratification, and hyperreflectivity of the RPE at the foveal centre. OCT: Optical coherence tomography; RPE: Retinal pigment epithelium

Optical coherence tomography (OCT) analysis in the right eye was within normal limits. Left eye OCT analysis revealed thickening of the inner retinal layers in the macular area (Figure 2). There was loss of stratification, along with opacification of the inner retinal layers. The foveal contour was maintained, with hyperreflectivity of the retinal pigment epithelium (RPE) at the centre. These changes indicate acute ischaemia. The inner retinal layers are supplied by the superficial capillary plexus, which is derived from the central retinal artery. CRAO causes hypoperfusion of this plexus, resulting in cellular swelling and extracellular oedema, leading to opacification and loss of stratification of the inner retinal layers. All these signs are indicative of acute CRAO. The extent of this ischaemic damage indicates a poor visual prognosis for the patient.

## Discussion

Sudden visual loss is an ophthalmic emergency. CRAO is one of the common aetiological factors and can lead to sudden, profound, and painless loss of vision. 

The treatment of CRAO is aimed at dislodging the thrombus or emboli that are causing the obstruction. Different methods to achieve this include ocular massage, anterior chamber paracentesis, oral administration of acetazolamide, inhalation of 95% oxygen and 5% carbon dioxide, rebreathing of expired CO_2_ in a bag, and retrobulbar vasodilators. The combination of massage and oral acetazolamide can decrease the intraocular pressure by 5 mm of Hg over a short period of time [6].

Anterior chamber paracentesis causes a sudden fall in intraocular pressure, which leads to improvement in retinal perfusion and sometimes dislodges the emboli. In a study conducted by Fieß et al., they concluded that paracentesis may not be effective but carries risks of intraocular infection and injury [7].

Wang et al. conducted a study to evaluate the safety and efficacy of ophthalmic artery branch retrograde intervention in the treatment of CRAO. They concluded that ophthalmic artery branch retrograde thrombolytic intervention was highly effective and safe in the treatment of CRAO, and it can be considered an alternative method for the delivery of therapeutic drugs in retinal vascular occlusive diseases [8].

Mehboob et al. conducted a study to determine the efficacy of yttrium aluminium garnet (YAG) laser embolysis in retinal artery occlusion. They found that if neodymium-doped yttrium aluminium garnet (Nd:YAG) embolysis is performed within six hours of the onset of symptoms, it is more effective in the management of fovea-threatening retinal artery occlusion compared to conventional medical management [9].

Chodnicki et al. conducted a study to determine the risk of stroke before and after CRAO and found an association between ischaemic stroke and CRAO. Overall, 5.3% of participants suffered an ischaemic stroke either before, during, or after the episode of CRAO. Of these, 2.3% had a stroke within 15 days before CRAO, 1.3% had a stroke at the same time as CRAO, and 1.7% had a stroke within 15 days after CRAO. The strokes associated with CRAO were also linked to an embolic aetiology [10].

In addition, Ratra and Dhupper conducted a study on the young Indian population to identify any systemic causes, such as hypertension or cardiac abnormalities, associated with CRAO. The study revealed that 65.6% of patients had a hypercoagulable state. Among these, 21.9% of patients had hyper-homocysteinemia, which was the most common cause. Moreover, 6.2% of patients had no systemic association or any other abnormality attributed to CRAO [11].

Furthermore, Bukhari et al. concluded that the incidence of stroke in young adults is rising globally. This is mainly due to an increase in patients with modifiable risk factors, such as hypertension, diabetes mellitus, obesity, and hypercholesterolemia. Other non-modifiable factors include migraine and a hypercoagulable state [12].

Anticoagulants used early in CRAO may reduce the volume of infarcted tissue by reducing the size of the thromboembolism and decreasing the extent of tissue loss. Oral anticoagulants (OACs) are recommended, as they might inhibit the formation of new arterial thromboses and reduce the risk of early recurrent thromboembolic stroke. In a study by Cools et al., discontinuation was defined as the cessation of OAC treatment for more than seven consecutive days. It was noted that the patients who discontinued treatment were more likely to be from the younger age group and to have a history of stroke or TIA. The main finding of this large, prospective real-world cohort was that patients who discontinued OAC treatment had worse clinical outcomes, with a higher chance of stroke. This study also highlights the need to educate patients in the younger age group with stroke risk factors and the need for adherence to anticoagulant therapy for long-term management [13].

In an article on CRAO by Tripathi et al., they discussed that patients diagnosed with CRAO, especially those in younger age groups, should be educated regarding the signs and symptoms of CRAO. The urgency of seeking immediate medical attention in the event of sudden vision loss should be emphasised, along with the recommendation to regularly check their vision by closing alternate eyes to detect any sudden visual decline. Patients should be advised about the necessity of undergoing a thorough examination by healthcare professionals, including eye specialists and possibly vascular specialists. Regular monitoring is essential to assess the patient's visual function, manage risk factors, and address potential complications. Hence, patients should be counselled appropriately regarding the importance and necessity of the same [14].

## Conclusions

In this study, the patient was young when he had his first stroke. He also had a history of diabetes mellitus, hyperlipidaemia, smoking, and alcohol consumption. He had discontinued anticoagulants on his own. Two weeks prior to this episode, he experienced a TIA episode, for which he did not seek any medical consultation. All these are risk factors, which contribute individually or collectively to the development of CRAO, with subsequent profound impairment of vision at a relatively young age.

This case highlights the importance of counselling and education for patients who have risk factors for stroke and retinal arterial occlusion. Patients should be counselled regarding the continuation of all prescribed medications, cessation of smoking and alcohol intake, and good control of all systemic diseases. The necessity of regular follow-up should be well emphasised to patients, especially in the younger age group, as both ocular and cerebral stroke can occur as they age.
